# 贝伐珠单抗联合培美曲塞加卡铂新辅助化疗方案对Ⅲa期肺腺癌手术安全性的影响

**DOI:** 10.3779/j.issn.1009-3419.2015.06.06

**Published:** 2015-06-20

**Authors:** 松亮 张, 鹏飞 徐, 成 袁, 伟 区

**Affiliations:** 510060 广州，中山大学肿瘤防治中心，华南肿瘤国家重点实验室 Sun Yat-sen University Cancer Center, State Key laboratory of South China, Guangzhou 510060, China

**Keywords:** 肺肿瘤, 贝伐珠单抗, 新辅助化疗, 安全性, Lung neoplasms, Bevacizumab, Neoadjuvant chemotherapy, Safety

## Abstract

**背景与目的:**

贝伐珠单抗联合化疗在晚期肺腺癌治疗中已取得较好疗效，本研究探讨贝伐珠单抗联合培美曲塞加卡铂方案作为Ⅲa期肺腺癌患者的新辅助方案对手术安全性的影响。

**方法:**

选择在中山大学肿瘤防治中心应用贝伐珠单抗联合培美曲塞加卡铂方案进行新辅助化疗的Ⅲa期肺腺癌患者25例，所有病例均在新辅助化疗2周期后，接受肺叶或者全肺叶切除加纵隔淋巴结清扫术。分析患者在化疗期间的毒性反应以及患者在围手术期间的并发症。

**结果:**

与新辅助化疗相关的3级-4级的不良事件包括3例疲劳事件、3例嗜中性白血球减少症和1例高血压。认为与贝伐珠单抗相关的不良事件包括2例鼻出血（1例1级、1例2级）和3例高血压（2例1级、1例3级）。在围手术期出现的并发症包括肺炎（2例）、支气管残端瘘（1例）、肺不张（2例）和心律失常（1例）。围手术期没有观察到既往贝伐珠单抗联合化疗中常见的出血事件、血栓事件以及伤口愈合问题的发生。

**结论:**

贝伐珠单抗联合培美曲塞加卡铂作为新辅助化疗方案是对于Ⅲa期肺腺癌患者来说是安全的，可以耐受的。

## Abstract (en)

**Background and objective:**

Bevacizumab has showed its efficacy in advanced non-squamous lung cancer. The aim of this study is to assess the safety of bevacizumab plus pemetrexed and carboplatin neoadjuvant chemotherapy in patients with lung adenocarcinoma.

**Methods:**

25 patients with Ⅲa lung adenocarcinoma undergoing lobectemy or pneumonectomy with mediastinal lymphadenectomy after induction bevacizumab (Bev) plus pemetrexed/carboplatin (PC) were selected. Toxicity of chemotherapy and postoperative complications were analyzed.

**Results:**

Grade 3 or 4 neoadjuvant-related adverse events included fatigue (3 patients), neutropenia (3 patients), hypertension (1 patient). The adverse events thought to be related to bevacizumab included epistaxis in 2 patients (grade 1: 1; grade 2: 1) and hypertension in 3 patients (grade 1: 2; grade 3: 1). Postoperative complications included pneumonia in 2 patients, bronchial stump insufficiency in 1 case, atelectasis in 2 cases, and arrhythmia in 1 case. Hemorrhage events, thromboembolic events and wound-healing problems were not observed in the perioperative period.

**Conclusion:**

The treatment modality of neoadjuvant Bev-PC appears to be safe and tolerant in patients withstage Ⅲa lung adenocarcinoma.

贝伐珠单抗是一种通过拮抗血管内皮生长因子来抑制血管生成的单克隆抗体。其作用主要表现在三个方面：①抑制新生或复发的肿瘤血管的生长。单药贝伐珠单抗几乎可以完全阻断肿瘤的生长和转移期间所必需新生血管的生长。另外，有研究^[1]^显示贝伐珠单抗可以对复发肿瘤内血管的生长产生持续的抑制。②使肿瘤内血管退化。在临床前模型中贝伐珠单抗可以引起80%的肿瘤血管退化^[2]^。③使肿瘤血管正常化。贝伐珠单抗可以使未成熟的肿瘤血管退化，减少血管渗透性，降低肿瘤组织内的压力^[3]^。正常化的血管使化疗药物容易到达，增加肿瘤细胞对化疗药物的敏感性，从而最大程度发挥化疗药物的抗肿瘤作用^[4]^。含贝伐珠单抗的联合化疗方案已经被应用于晚期肺腺癌的一线治疗中。多项的大型随机临床研究结果^[5-8]^已经在一定程度上揭示了其在晚期非鳞癌中的有效性及安全性。但关于其新辅助化疗的研究报道甚少。我们通过总结25例Ⅲa肺腺癌患者接受联合贝伐珠单抗新辅助化疗期间毒性反应以及围手术期并发症，探讨其作为新辅助化疗方案对手术安全性的影响。

## 1 资料与方法

### 1.1 病例选择

选取在中山大学肿瘤防治中心接受过贝伐珠单抗联合培美曲塞加卡铂新辅助化疗后接受手术的肺腺癌患者25例。男性12例，女性13例，中位年龄49岁（30岁-65岁）。所有患者术前均接受贝伐珠单抗（7.5 mg/kg, d1）联合培美曲塞（500 mg/m^2^, d1）加卡铂方案的新辅助化疗。在新辅助化疗后3周-4周内接受全肺叶切除/肺叶切除/联合肺叶切除加上纵隔淋巴结清扫术。其中有7例患者接受全肺叶切除，其余患者接受肺叶或者联合肺叶切除。患者的详细临床基本特征见表 1。

**1:** 患者的临床特征（*n*=25） Clinical charateristics of patients (*n*=25)

Chacrateristic		*n*	Percentage
Age (yr)	Middle age/Range	49/30-65	-/-
Gender	Male/Female	12/13	48/52
Surgery	Pneumonectomy/Lobectomy	7/18	28/72
Location	Centrl/Peripheral	5/20	20/80
Smoking	Never/Former/Current	8/3/14	32/12/56

### 1.2 观察指标

总结所有患者在接受贝伐珠单抗联合培美曲塞加卡铂新辅助化疗期间的毒性反应（①非血液学毒性反应：出血事件、高血压、疲劳、恶心等；②血液学毒性反应：中性粒细胞减少、贫血、血小板减少等），以患者在围手术期间的并发症（包括出血事件、血栓事件、肺炎、肺不张、支气管残端瘘、伤口愈合延迟等）情况。化疗期间的不良事件分级依据美国国立癌症研究所制定的通用毒性评价标准[National Cancer Institute Common Toxicity Criteria (version 3.0)]。

## 2 结果

### 2.1 化疗及毒性反应

贝伐珠单抗联合化疗21 d为1个周期，共2个周期。在第1周期所有患者接受维生素B12肌肉注射，化疗间隔期间口服多元维生素片。在新辅助化疗期间，无患者因为毒性反应导致化疗延期或者停止化疗，观察到的非血液学不良事件有：鼻出血（2例，8%）、高血压（3例，12%）、疲劳（6例，24%）、恶心（1例，4%）、腹泻（1例，4%）、乏力（2例，8%）。血液学不良反应方面，主要为嗜中性粒细胞减少（5例，20%）、贫血（2例，8%）、血小板减少（1例，4%）。其中3级-4级不良事件有7例，包括疲劳3例（12%）、嗜中性白血球减少3例（12%）和高血压1例（4%）。而认为与贝伐珠单抗相关的不良事件包括2例鼻出血（8%，1例1级；1例2级）和3例高血压（12%，2例1级；1例3级）。未发生3级以上的出血事件，未观察到血栓事件发生（表 2）。

**2:** 化疗的毒性反应 Toxicity of chemotherapy

Adverse events	*n*	Grade 3 or 4
Nonhematologic		
Epistaxis	2	0
Hypertension	3	1
Fatigue	6	3
Nausea	1	0
Diarrhea	1	0
Asthenia	2	0
Hematologic		
Neutropenia	6	3
Anemia	2	0
Thrombocytopenia	1	0

### 2.2 围手术期并发症

观察到的短期术后并发症（术后30 d内）主要有：肺炎（2例）、肺不张（2例）和心律失常（1例）。长期术后并发症（术后30 d后）有1例支气管残端瘘。围手术期未观察到相关出血事件、血栓事件。术后未有患者因接受贝伐珠单抗联合化疗而致伤口愈合延迟。围手术期未出现患者死亡。

## 3 讨论

随着贝伐珠单抗的推广运用，其安全性也受到广泛关注。其在晚期非鳞癌中的有效性及安全性已经被证实，但关于其在新辅助化疗方面的安全性却少有报导。我们通过分析25例患者在接受贝伐珠单抗联合化疗期间的毒性反应以及后续围手术期的并发症，来评估贝伐珠单抗联合化疗在新辅助化疗方面的安全性。

出血事件是贝伐珠单抗应用中较常见的不良事件，Johnson等^[9]^在晚期肺癌一线化疗的临床研究结果表明，中央型肺癌以及组织类型是鳞癌的患者，其咯血事件的发生率明显高于其余患者，此结果导致鳞癌以及中央型肺癌的患者被排除在贝伐珠单抗的临床研究之外。本组患者的5例中央型肺癌的患者在化疗期间未观察到相关的咯血事件，仅有2例鼻出血，均为1级-2级，不影响贝伐珠单抗的用量。其原因可能与本组患者的病变较早期、肿瘤负荷小，新辅助化疗周期少（2个周期）以及体能状态评分低（0分-1分）有关。化疗期间观察到的3级-4级毒性反应有3例疲劳、3例嗜中性白血球减少以及1例高血压。其余化疗毒性反应，包括恶心、腹泻、乏力、贫血以及血小板减少，均反应轻微，患者表现出良好的耐受性。

在以往结直肠癌新辅助化疗的临床研究中，应用贝伐珠单抗联合方案新辅助化疗后的患者，在围手术期较常见的严重不良事件是胃肠道穿孔、伤口愈合延迟、出血以及动脉栓塞，但发生率都较低，均不大于2%^[10-12]^。在应用贝伐珠单抗新辅助化疗后，接受手术的结直肠癌患者伤口愈合延迟的发生率明显高于对照组。而本文中的25例患者的伤口在术后并未进行特殊处理，均在7 d-9 d内愈合拆线，并未出现因贝伐珠单抗联合化疗的应用而出现愈合延迟。其原因可能与患者手术时间安排有关，25例患者均在贝伐珠单抗应用后的3周-4周后进行手术，错开了手术与贝伐珠单抗应用的时间。其余较常见的严重并发症，如胃肠道穿孔、出血和动脉栓塞在本研究中没有出现。而观察到的其余围手术并发症，如肺炎、肺不张、心率失常发生率与既往非小细胞肺癌新辅助化疗研究中的报导无明显差异^[13]^。此组患者中仅有1例患者术后出现支气管瘘，经保守治疗后治愈，其发生原因是否与贝伐珠单抗导致的愈合困难有关，尚不能定论。

总的来说，贝伐珠单抗联合培美曲塞加卡铂新辅助化疗的毒性反应及围手术期并发症对于Ⅲa期肺腺癌患者来说是可以耐受的，可作为Ⅲa期肺腺癌患者的新辅助化疗方案，但其疗效还有待进一步的探索。
